# Health Perception among Female COVID-19 Patients. Comment on Fernández-de-las-Peñas et al. Female Sex Is a Risk Factor Associated with Long-Term Post-COVID Related-Symptoms but Not with COVID-19 Symptoms: The LONG-COVID-EXP-CM Multicenter Study. *J. Clin. Med.* 2022, *11*, 413

**DOI:** 10.3390/jcm11112999

**Published:** 2022-05-26

**Authors:** Blanca Ayuso García, Eva Romay Lema, Ramón Rabuñal Rey

**Affiliations:** Infectious Diseases Unit, University Hospital Lucus Augusti, Rua Doutor Ulises Romero, s/n, 27003 Lugo, Spain; eva_rl88@hotmail.com (E.R.L.); ramon.rabunal.rey@sergas.es (R.R.R.)

We read with interest the original paper by Fernández-de-las-Peñas et al. [1], in which they report the results of a multicentre, cross-sectional study aimed at distinguishing the symptoms of COVID-19 in men and women both at presentation and in the long term. This has been also addressed by other research teams [2,3].

In the same vein, our Unit conducted a cross-sectional study in patients infected with SARS-CoV-2 during the first pandemic wave, who were contacted 10 months after the infection to assess their involvement after acute infection and the factors related to this fact. A telephone survey was administered using the EQ-5D-5L and C19-YRS scales. EQ-5D-5L is a validated tool for the assessment of health-related quality of life (HRQOL), and its results for different populations are tabulated and available for consult [4]. C19-YRS is a specific tool designed for the investigation and quantification of post-COVID syndrome, which allows for the comparison of symptoms before and after the acute infection [5].

We included all patients with a RT-PCR diagnosis of SARS-CoV-2 infection, both hospitalized and ambulatory [6]. Statistical analysis was conducted using Mann–Whitney test for quantitative variables and Pearson’s chi-squared test for categorical variables. Out of 745 infected patients, 44 died and 93 were excluded due to dementia, after which 443 answers were obtained (answer rate 72.9%). The mean age was 54 ± 16 years, 38.4% of the patients were male, and 42.9% had any previous condition. The hospitalization rate was 19.6%, and 2.3% needed admission to an intensive care unit (ICU).

Female patients were younger than males (age > 65 years in 23.1% vs. 33.5%, *p* = 0.016) and had lower rates of comorbidity (34.8% vs. 55.9%; *p* < 0.001) and hospital (15.8% vs. 25.9%; *p* = 0.009) and ICU (1.1% vs. 4.1%; *p* = 0.042) admission.

Despite this, women had significantly higher rates of pain (17.9% vs. 5.9%; *p* < 0.001) and anxiety/depression (27.8% vs. 17.6%; *p* = 0.014) than men when using EQ-5D-5L, and female sex was an independent risk factor for lower scores on the HRQOL perception scale. When C19-YRS was used, the rates of pain of new onset (22.0% vs. 8.8%; *p* < 0.001), airway complaints (15.0% vs. 7.1%; *p* = 0.012), focusing difficulties (20.9% vs. 12.4%; *p* = 0.022), and short-term memory impairment (23.5% vs. 15.9%; *p* = 0.020) were also significantly higher among women. Only swallowing difficulties were more frequent in men than women (5.3% vs. 1.8%; *p* = 0.043).

As Fernández-de-las-Peñas and colleagues note, our results also suggest that female sex might be related to a worse HRQOL perception in terms of symptoms after the acute infection, even if the disease is less severe during that phase. This has been also observed in other conditions like chronic fatigue [7], connective tissue disorders [8], diabetes mellitus [9], or cardiovascular diseases like atrial fibrillation [10]. This effect seems to be independent of the severity of these illnesses [1,6,8,10,11]. Additionally, a worse overall perception of health has been noted among women in healthy general population samples [12,13,14], independent of age and educational or income status [11,14,15]. Moreover, women also attain lower scores in previous EQ-5D reports [11,14,16], so these differences might be related to factors other than COVID-19.

As a conclusion, since differences in health self-perception in women remain observable irrespective of biological phenomena and socio-economical aspects other than sex itself, there are probably additional social and personal factors intrinsic to women that contribute to this worse health experience. This should make us reconsider the current focus on women’s health and HRQOL issues. Additionally, to better ascertain both the presence of symptoms and the HRQOL in women, validated tools and scales may be more useful, since they allow for comparing results with contemporary and historic cohorts.
